# Impact of Forced Swimming Stress on Serum Adiponectin and Endothelin-1 Levels in Wistar Rats: Comparative Analysis of Dietary Effects

**DOI:** 10.7759/cureus.58405

**Published:** 2024-04-16

**Authors:** Almira Hadžović-Džuvo, Amina Valjevac, Asija Začiragić, Alen Kekić, Orhan Lepara

**Affiliations:** 1 Department of Human Physiology, Faculty of Medicine, University of Sarajevo, Sarajevo, BIH

**Keywords:** forced swimming, exercise, rats, high fat diet, endothelin1, adiponectin

## Abstract

Aim

This study aimed to assess the impact of forced repeated swimming stress on serum adiponectin and endothelin-1 levels in Wistar rats, comparing the effects between those fed a standard diet and those on a high-fat diet.

Methods

Twenty adult male Wistar rats were divided into two dietary groups: a standard food diet group (NFD, n=10) and a high-fat diet group (HFD, n=10). Both groups underwent daily forced swimming stress for six days, with durations increasing from 5 to 30 minutes. The protocol finished in an acute bout of swimming exercise on the seventh day with a duration of 40 minutes. Adherence to ethical guidelines was strictly maintained, and serum adiponectin and endothelin-1 levels were measured pre- and post-exercise using the ELISA method.

Results

Before swimming, the mean adiponectin levels were 4.30±1.50 ng/mL in the NFD group and 3.53±0.70 ng/mL in the HFD group. Post-exercise, these levels significantly decreased to 2.4±0.84 ng/mL (p=0.003) and 1.58±0.23 ng/mL (p=0.001), respectively. Endothelin-1 levels also showed significant decreases from 0.86 pg/mL (0.74-0.87) to 0.49 pg/mL (0.43-0.62) (p=0.003) in the NFD group, and from 0.89 pg/mL (0.86-0.93) to 0.69 pg/mL (0.60-0.75) (p=0.027) in the HFD group after swimming.

Conclusion

The study highlighted the significant effects of forced swimming stress on lowering serum adiponectin and endothelin-1 levels in Wistar rats, with more pronounced decreases observed in rats on a high-fat diet. The results of the study suggest the potential of exercise as a crucial component of strategies aimed at managing obesity and improving cardiovascular health, emphasizing the interaction between physical stress and dietary factors on metabolic and cardiovascular biomarkers.

## Introduction

The global obesity epidemic and its associated metabolic disorders, such as type 2 diabetes, cardiovascular disease (CVD), hypertension, and several cancers, pose significant public health challenges worldwide [1]. Regular exercise has been identified as a crucial method for enhancing overall health and reducing the risk of these conditions. Among the strategies to combat obesity, exercise emerges not only as a preventive measure but also as a therapeutic intervention, highlighting its importance in health enhancement and disease risk reduction [2]. Adipocytokines, particularly resistin, leptin, and adiponectin, play a crucial role in the onset of obesity and related metabolic diseases. Notably, adiponectin is distinguished by its positive regulatory effects on obesity and associated metabolic syndromes, influencing various physiological processes including inflammation, insulin sensitivity, and metabolic flexibility [3]. However, the impact of exercise on adiponectin regulation has shown inconsistent results, indicating the complexity of exercise-induced physiological responses [4-6]. This underscores the nuanced interplay between lifestyle factors and metabolic health, further complicated by the influence of dietary factors, especially high-fat diets, on metabolic regulation [2, 7, 8]. The genetics of obesity also underline the multifactorial nature of this condition, emphasizing the need for multifaceted interventions [1]. The dysregulation of adipocytokines, such as resistin, leptin, and especially adiponectin, plays a pivotal role in the pathophysiology of metabolic diseases. Adiponectin shows promise as a therapeutic target due to its beneficial effects on metabolism and inflammation [3]. Additionally, the role of diet, particularly high-fat diets, complicates the interplay between exercise, adiponectin regulation, and endothelial function, highlighting the need for integrated lifestyle interventions [7-9]. Moreover, endothelin-1 (ET-1), a potent vasoconstrictor, has been implicated in the pathophysiology of obesity, with increased levels in the blood and tissues associated with adverse metabolic outcomes [10]. Regular exercise is posited as an effective means to reduce ET-1 concentrations and, by extension, lower overall cardiovascular disease risk [11]. The exercise-induced redistribution of blood flow, mediated by ET-1's action on vascular smooth muscle, highlights the potential of exercise to improve endothelial function and reduce cardiovascular risk, particularly in overweight and obese populations [10, 11]. Given these considerations, the present study aimed to investigate the effects of short-term forced swimming stress on serum adiponectin and endothelin-1 levels in rats under high-fat and non-fat diet conditions. This study seeks to contribute valuable insights into the complex interactions between exercise, diet, and key metabolic and vascular markers, emphasizing the importance of personalized approaches in managing obesity and metabolic syndrome [2-4, 10, 11].

## Materials and methods

Animal model and experimental design

This study was conducted on 20 adult male Wistar rats, maintained in a temperature-controlled environment at 20°C, with consistent humidity and subjected to a 12-hour light-dark cycle. The handling and care of the animals adhered strictly to the ethical guidelines for the use of laboratory animals, as approved by the Local Committee of Science and Research Ethics, University of Sarajevo, Bosnia and Herzegovina.

The rats were allocated into two distinct groups:

I) Control group, fed a standard diet (NFD, n=10)

II) Experimental group, fed a high-fat diet (HFD, n=10)

The standard chow diet comprised 22.4% fat, 20.5% protein, and 57.1% carbohydrates, yielding an energy content of 294 kcal/100g. Conversely, the high-fat diet contained 46.8% fat, 32.1% protein, and 21.1% carbohydrates, totaling an energy content of 576.8 kcal/100g. Water was made available ad libitum. Both groups fed assigned diet from the beginning until the end of the study.

Exercise protocol

Prior to the main exercise intervention, an acclimatization period was instituted, wherein rats underwent daily swimming sessions to familiarize them with water and mitigate stress responses to swimming. Both groups were subjected to daily forced swimming exercises between 10:00 AM and 11:00 AM for six days, with the session duration incrementally increasing from 5 minutes on the initial day to 30 minutes by the sixth day. The swimming was conducted in plastic tanks (90 cm width × 120 cm depth), filled with tap water maintained at 25°C and a depth of 40 cm, ensuring no more than two rats swam concurrently. On the seventh day, an acute bout of swimming exercise was performed, lasting 40 minutes until exhaustion, under identical conditions to the adaptation period. Body weight measurements were taken at the beginning of the adaptation period and following the final swimming session.

Blood collection and biomarker analysis

Blood samples were collected immediately before and after the final swimming session from the tail vein, following thorough cleansing and drying of the tail. Serum concentrations of adiponectin and endothelin-1 were quantified utilizing the ELISA method, employing a STAT FAX 2100 machine (USA) at the Department of Physiology and Department of Biochemistry, Faculty of Medicine, University of Sarajevo. The Alpco ELISA kits (Alpco, Salem, NH, USA) were used for the analyses.

Statistical analysis

Data analysis was conducted using IBM SPSS Statistics version 21.0 (IBM Corp., Armonk, NY, USA), with results presented as mean ± standard deviation, and median with interquartile range (25th-75th percentiles). The normality of distribution was evaluated using the Shapiro-Wilk test. Group comparisons for independent and dependent variables were conducted using Student's t-test, paired t-test, and the Wilcoxon test, as appropriate. A p-value of less than 0.05 was deemed indicative of statistical significance.

## Results

The value of adiponectin in the total sample before swimming training was 4.02±1.31 ng/mL but after swimming training, it dropped significantly to a value of 2.11±0.78 ng/mL (p<0.001). The value of endothelin-1 in the total sample before swimming training was 0.87 pg/mL (0.77-0.91) and after swimming training it significantly decreased to a value of 0.62 pg/mL (0.44-0.69) (p<0.001). The value of body mass before and after swimming training did not differ significantly (p=0.062) (Table 1).

**Table 1:** Mean values of body weight, adiponectin and endothelin-1 concentration in rats before and after exercise

Variables	Before exercise (n=20)	After exercise (n=20)	p
Body mass (g)	277.94±55.42	286.46±67.14	0.062
Adiponectin (ng/mL)	4.02±1.31	2.11±0.78	< 0.001
Endothelin-1 (pg/mL)	0.87 (0.77-0.91)	0.62 (0.44-0.69)	< 0.001

The value of adiponectin after swimming training in the NFD group was 2.4±0.84 ng/mL, while in the HFD group, it was 1.58±0.23 ng/mL, and the determined difference was significant (p=0.036). A significant difference was found in ET-1 values before swimming training between NFD and HFD groups [0.80±0.10 vs. 0.90±0.03 (p=0.037)], as well as in ET-1 values after swimming training [0.52±0.11 vs. 0.67±0.11 (p=0.049)]. The value of adiponectin before swimming training between the examined groups was not significant (p=0.263) (Table 2).

**Table 2:** Mean serum adiponectin and endothelin-1 concentration in rats on a standard food diet and high-fat diet before and after exercise NFD - standard food diet group HFD - high-fat diet group

Variables	NFD (n=10)	HFD (n=10)	p
Adiponectin before exercise (ng/mL)	4.30±1.50	3.53±0.70	0.263
Adiponectin after exercise (ng/mL)	2.4±0.84	1.58±0.23	0.036
Endothelin-1 before exercise (pg/mL)	0.80±0.10	0.90±0.03	0.037
Endothelin-1 after exercise (pg/mL)	0.52±0.11	0.67±0.11	0.049

**Figure 1:**
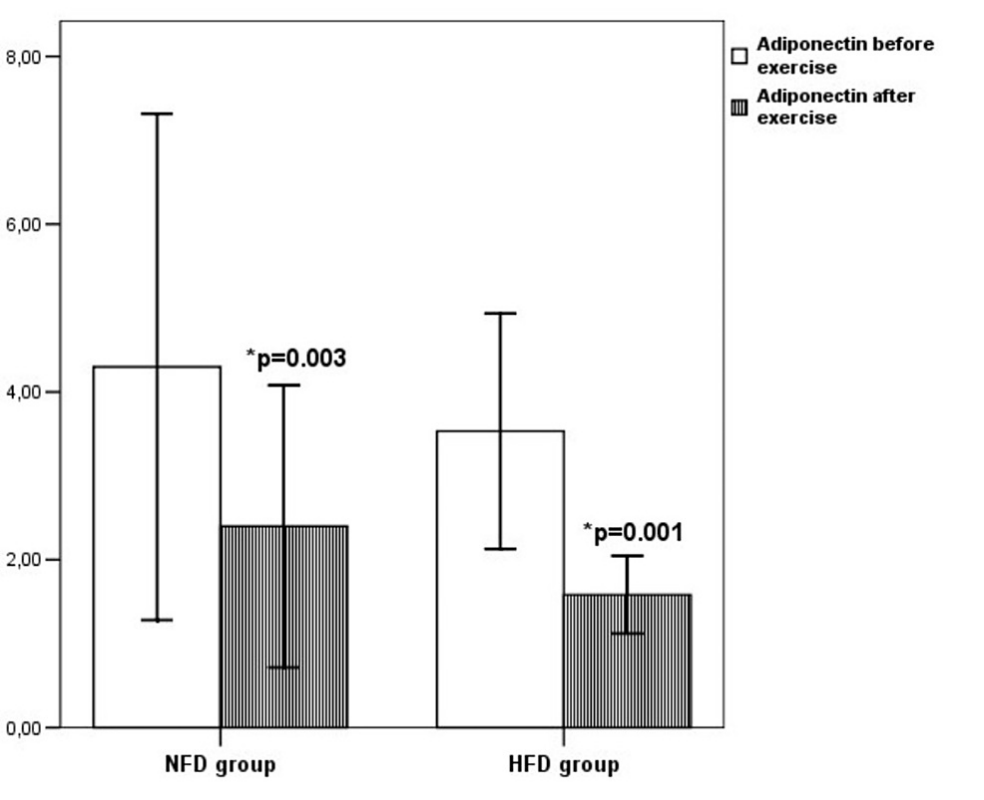
Serum adiponectin concentration in rats on a standard food diet and high-fat diet before and after exercise *in comparison to the adiponectin concentration before exercise in the same group of rats NFD - standard food diet HFD - high-fat diet

Mean concentration of adiponectin in the NFD group before swimming was 4.30±1.50 ng/mL, but after swimming training it significantly decreased to a value of 2.4±0.84 ng/mL (p=0.003). Mean concentration of adiponectin in the HFD group before the swimming training was 3.53±0.70 ng/mL, but after the swimming training it significantly decreased to a value of 1.58±0.23 ng/mL (p=0.001) (Figure 1).

**Figure 2:**
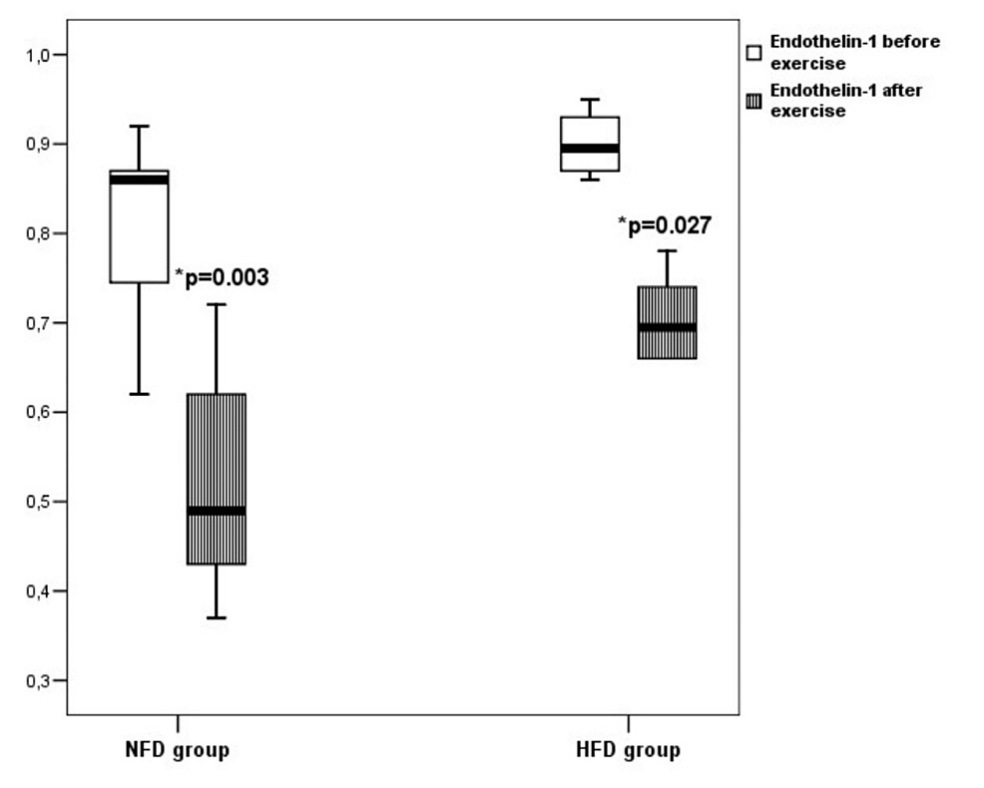
The concentration of endothelin-1 in rats on a standard food diet and high-fat diet before and after exercise *in comparison to the endothelin-1 concentration before exercise in the same group of rats NFD - standard food diet HFD - high-fat diet

The concentration of endothelin-1 in the NFD group before swimming training was 0.86 pg/mL (0.74-0.87) and after swimming training it significantly decreased to a value of 0.49 pg/mL (0.43-0.62) (p=0.003). The concentration of endothelin-1 in the HFD group before swimming training was 0.89 pg/mL (0.86-0.93), but after swimming training, it significantly dropped to a value of 0.69 pg/mL (0.60-0.75) (p=0.027) (Figure 2).

## Discussion

Our study's findings on the impact of short-term forced swimming stress on serum adiponectin and endothelin-1 levels in rats offer significant insights into the physiological responses to exercise and dietary conditions. The observed decrease in adiponectin levels post-exercise, particularly pronounced in the high-fat diet group, may reflect acute metabolic adjustments and underscores the importance of considering dietary composition in exercise interventions [2, 7, 8]. Furthermore, the reduction in endothelin-1 levels post-exercise suggests beneficial effects on vascular function and highlights the potential of physical activity in reducing cardiovascular risk, especially in overweight and obese populations [10, 11].

The significant decrease in adiponectin levels following swimming stress, as observed in both normal and high-fat diet (NFD and HFD) groups, aligns with inconsistent findings reported in the literature regarding exercise-induced adiponectin regulation [4]. While exercise is generally considered beneficial for metabolic health, our findings suggest that the type, intensity, and duration of physical activity, alongside dietary factors, can differentially influence adiponectin dynamics [12-14]. This reduction could be attributed to acute physiological stress responses to exercise, which may transiently lower adiponectin concentrations, potentially due to increased energy expenditure and metabolic adjustments [12].

Moreover, the observed decrease in endothelin-1 levels post-exercise in both diet groups provides evidence for the beneficial effects of regular physical activity on vascular function, particularly in lowering ET-1-mediated vasoconstrictor tone [10, 11]. This finding is significant, considering the role of ET-1 in the pathophysiology of obesity and its contribution to cardiovascular disease risk [10]. Regular exercise, as exemplified by swimming stress in this study, may thus serve as a key intervention to improve endothelial function and reduce cardiovascular risk, particularly in overweight and obese populations [11].

The comparison between NFD and HFD groups before and after exercise revealed significant differences in both adiponectin and endothelin-1 levels, highlighting the influence of dietary composition on exercise-induced physiological responses [2, 7, 8]. The more pronounced decrease in adiponectin levels in the HFD group post-exercise may reflect the compounded effects of a high-fat diet on adiponectin regulation, exacerbating the challenges in managing obesity and related metabolic disturbances. Similarly, the differential response in endothelin-1 levels suggests that diet quality can modulate the beneficial effects of exercise on endothelial function, emphasizing the need for integrated dietary and physical activity interventions for optimal metabolic health outcomes.

Our findings underscore the importance of considering individual variability in exercise and dietary interventions for obesity and metabolic syndrome. The complex responses of adiponectin and endothelin-1 to exercise, influenced by dietary factors, suggest that personalized approaches may be necessary to optimize therapeutic outcomes. Furthermore, the beneficial effects of exercise on endothelial function, as indicated by decreased ET-1 levels, highlight the potential of physical activity as a non-pharmacological strategy to mitigate cardiovascular risk in obese populations [15].

## Conclusions

Future research is needed to elucidate the long-term impacts of different types and intensities of exercise on adiponectin and endothelin-1 levels, particularly in relation to dietary patterns. Understanding the mechanisms underlying the observed changes in adiponectin and endothelin-1 in response to exercise and diet will be crucial in developing targeted interventions for improving metabolic health and reducing the burden of obesity-related diseases.

Our study suggests that tailored exercise programs, taking into account both the type and intensity of physical activity, as well as dietary habits, could be beneficial for individuals aiming to improve metabolic health and reduce cardiovascular risk. For instance, individuals following high-fat diets may need to adjust their exercise routines to counteract potential reductions in adiponectin levels, while also reaping the vascular benefits of physical activity.

This study contributes to the growing body of evidence on the physiological effects of exercise on metabolic and cardiovascular health markers. The observed changes in adiponectin and endothelin-1 levels following swimming stress in rats highlight the potential of exercise as a key component of integrated strategies for managing obesity and enhancing cardiovascular health.
